# Regulation of Programmed Ribosomal Frameshifting by Co-Translational Refolding RNA Hairpins

**DOI:** 10.1371/journal.pone.0062283

**Published:** 2013-04-29

**Authors:** Che-Pei Cho, Szu-Chieh Lin, Ming-Yuan Chou, Hsiu-Ting Hsu, Kung-Yao Chang

**Affiliations:** Institute of Biochemistry, National Chung-Hsing University, Taichung, Taiwan, Republic of China; Kantonal Hospital St. Gallen, Switzerland

## Abstract

RNA structures are unwound for decoding. In the process, they can pause the elongating ribosome for regulation. An example is the stimulation of -1 programmed ribosomal frameshifting, leading to 3′ direction slippage of the reading-frame during elongation, by specific pseudoknot stimulators downstream of the frameshifting site. By investigating a recently identified regulatory element upstream of the SARS coronavirus (SARS-CoV) −1 frameshifting site, it is shown that a minimal functional element with hairpin forming potential is sufficient to down-regulate−1 frameshifting activity. Mutagenesis to disrupt or restore base pairs in the potential hairpin stem reveals that base-pair formation is required for−1 frameshifting attenuation *in vitro* and in 293T cells. The attenuation efficiency of a hairpin is determined by its stability and proximity to the frameshifting site; however, it is insensitive to E site sequence variation. Additionally, using a dual luciferase assay, it can be shown that a hairpin stimulated +1 frameshifting when placed upstream of a +1 shifty site in yeast. The investigations indicate that the hairpin is indeed a cis-acting programmed reading-frame switch modulator. This result provides insight into mechanisms governing−1 frameshifting stimulation and attenuation. Since the upstream hairpin is unwound (by a marching ribosome) before the downstream stimulator, this study’s findings suggest a new mode of translational regulation that is mediated by the reformed stem of a ribosomal unwound RNA hairpin during elongation.

## Introduction

Sequence complementarities and three-nucleotide based genetic codes in messenger RNA (mRNA) imbue interesting features for translation. These include a) an intra-molecular duplex (formed via sequence complementarities) having to be unwound for decoding, and b) one of three potential reading-frames having to be maintained for faithful protein synthesis. The ribosome possesses helicase activity that allows for the unwinding of RNA duplexes [1], [2] while reading-frame maintenance is closely coupled with translocation [3]. However, unwinding of specific RNA structures can pause or stall the ribosome for further elongation regulation [4]. In particular, specific mRNA signals can program a ribosome to switch reading-frames during elongation, with the ribosome slipping backward (toward the 5′-direction) or forward (toward the 3′-direction) by a single nucleotide. It then continues translation in the new−1 or +1 reading-frame. Such−1 or +1 programmed ribosomal frameshifting (PRF) has been characterized in prokaryotes and eukaryotes and is usually related to specific cellular functions [5].

A slippery sequence (XXXYYYZ) and optimally placed downstream stimulator structures on mRNA are the two *in-cis* elements required for efficient eukaryotic−1 PRF [6]. However, the precise−1 PRF stimulation mechanism remains unclear [7]. Most models of−1 PRF stimulation propose that a specific structural or mechanical feature of the stimulator resists the unwinding activity of ribosomal helicases [2]. This is done either passively by serving as a roadblock to pause ribosomal movement or actively by creating tension/strain to communicate with transfer RNA (tRNA)-mRNA linkages to destabilize the P site codon-anticodon helix in the 0-frame (XXY). It also eventually facilitates re-pairing of tRNA with the−1 frame mRNA (XXX) [2], [3], [8], [9], [10].

Other factors have been suggested in the modulation of frameshifting efficiency [5]. In particular, an optimally placed internal Shine-Dalgarno (SD) sequence in prokaryotic mRNA may serve as a −1 PRF stimulator by pairing with the anti-SD element in 16S ribosomal RNA (rRNA) of the 70S ribosome [11]. In addition, −1 frameshifting efficiency can be affected positively or negatively by flanking sequences upstream of a slippery site [12], [13]. Previously, we identified a 170-nucleotide RNA element (ATT), upstream of the−1 PRF slippery site of SARS-CoV mRNA, capable of down-regulating viral−1 PRF [14]. Recently, ATT was shown to optimize viral replication and was suggested to act by causing a fraction of the elongating ribosome to fall-off in front of the ATT [15]. Understanding how−1 PRF attenuation is achieved would not only shed light on how a −1 PRF stimulator promotes−1 frameshifting and provide insight into the mechanism governing reading-frame control, but also may have potential for antiviral applications because−1 PRF efficiency is crucial for the replication of several human viral pathogens, including HIV and SARS coronavirus (SARS-CoV) [15], [16].

Here, we identify a minimal element in SARS-CoV ATT as the major determinant of−1 PRF attenuation function. Additionally, we show that attenuation efficiency is not sensitive to E site sequence variation, suggesting flanking-sequences effect is not the main cause of attenuation. We further demonstrate that this minimal element acted through a hairpin form with attenuation efficiency determined by hairpin stability and spacing to the slippery site. Importantly, this potential hairpin also enhanced +1 frameshifting in yeast, indicating that in addition to being a −1 frameshifting attenuator, it can serve as a +1 frameshifting stimulator. Together, these results indicate that the upstream RNA hairpin functions as a cis-acting RNA motif in programmed frameshifting regulation. Finally, our findings also indicate base-pair reformation involving the terminal sequences of the 3′-half of the hairpin stem as being crucial for attenuation. This implies the existence of a refolding hairpin stem in close proximity to the ribosomal E site.

## Results

### The Minimal Upstream Attenuation Determinant May Act through a Hairpin Form

**Figure 1:**
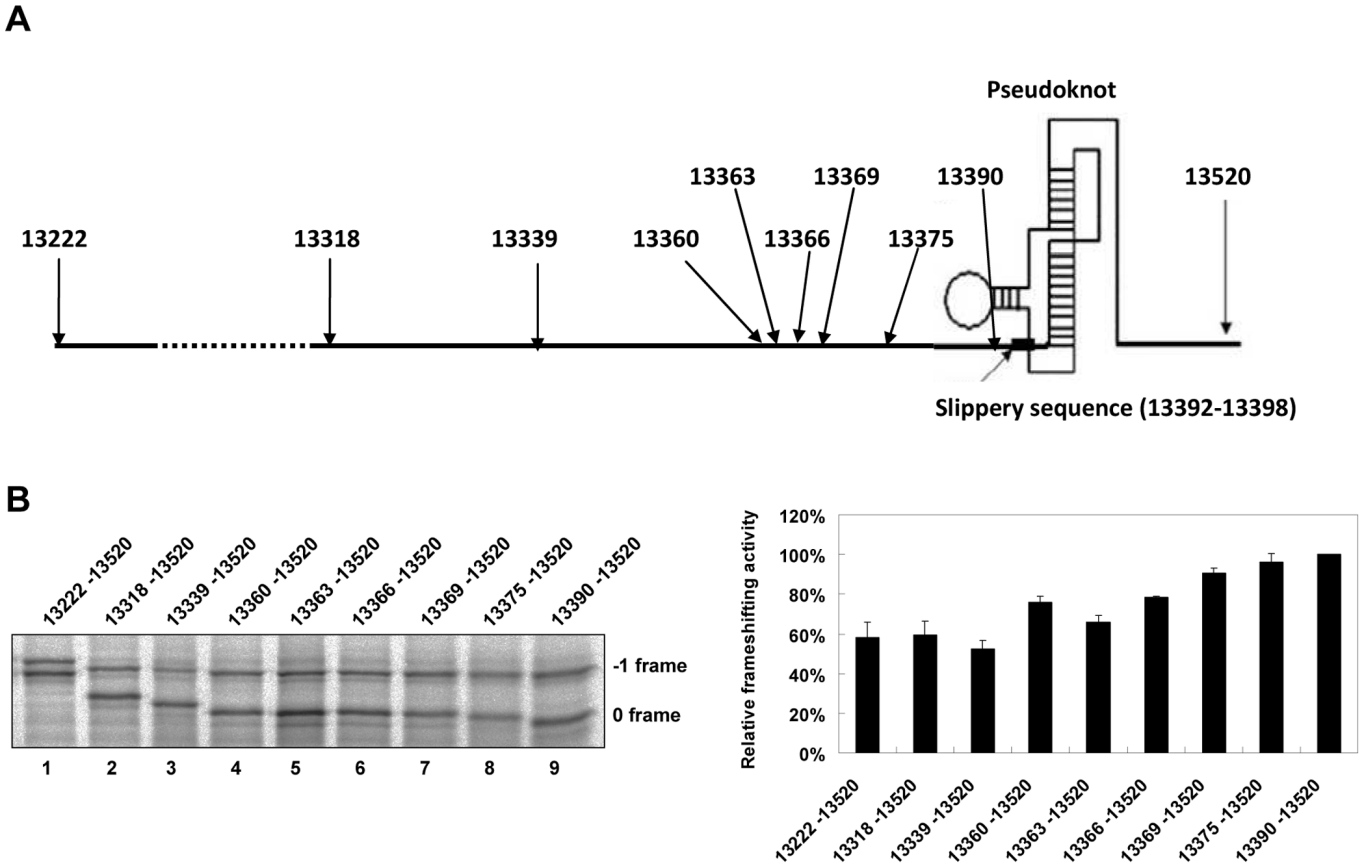
Deletion analysis identifies the minimal attenuator element in SARS-CoV viral sequence. (A) Schematic drawing of SARS-CoV viral genomic region spanning−1 PRF signal and its upstream element covering sequences 13222–13520 of SARS-CoV. Each of different 5′-viral deletion fragments (annotated by arrow) was cloned into a dual-luciferase−1 PRF reporter with a shortened −1 reading-frame. (B) *In vitro* −1 PRF assays by SDS-PAGE analysis of ^35^S methionine-labeled translation products for reporter constructs containing different 5′ deletions of the ATT element (left) and the relative frameshifting activities of different deletion mutants (right). The two major bands in each lane correspond to 0 (lower) and −1 (higher) frame translation products. The relative extent of frameshifting was determined as the ratio between each mutant and the 13390–13520 containing reporter construct (being treated as 100%). The value for each construct is presented as mean±SD (error bars) of triplicate experiments.

**Figure 2:**
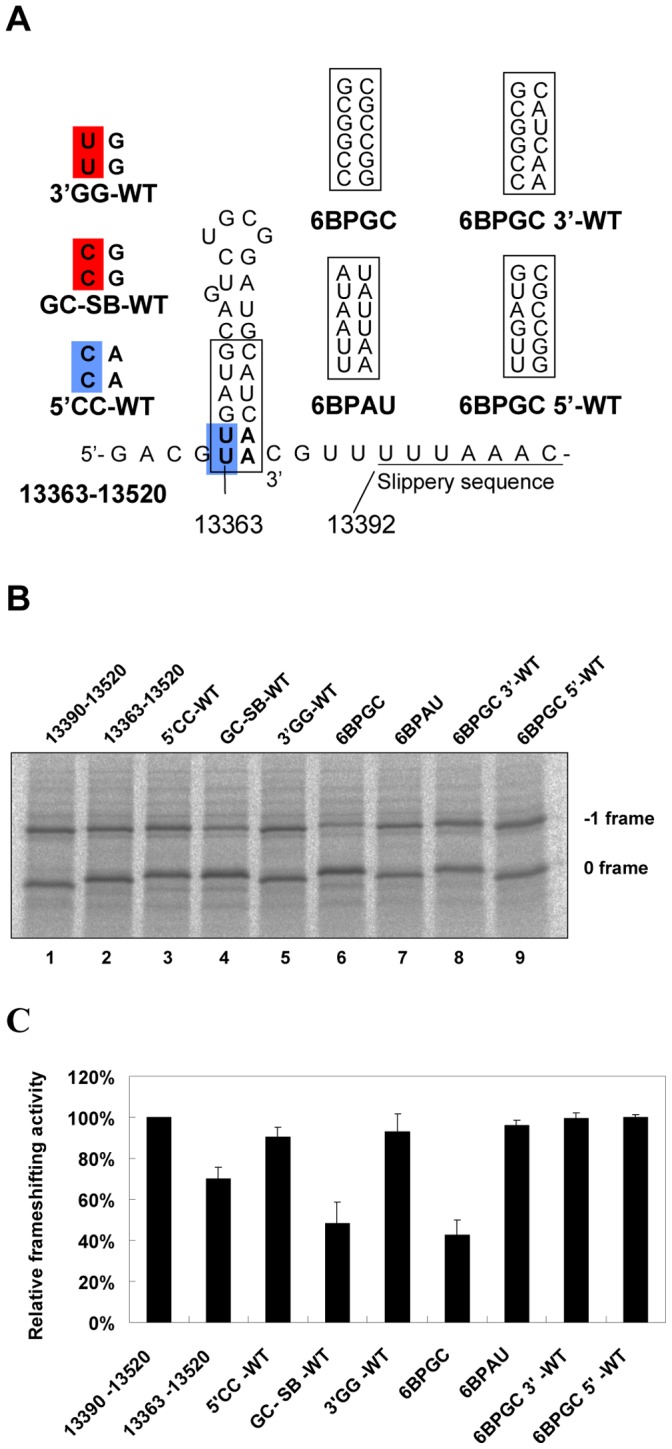
Base-pairing formation of the predicted hairpin stem is required for efficient −1 PRF attenuation activity. (A) Illustration of constructs with different base-pairing schemes at the lower stem of the predicted hairpin. For each mutant, the nucleotide composition after mutation is boxed or boldly typed. Two nucleotides, 27 nucleotides upstream of the 0-frame E site and those involved in terminal stem base-pairing formation, are colored for comparison. (B) *In vitro* −1 PRF assays by SDS-PAGE analysis of ^35^S methionine-labeled translation products for constructs with different base-pairing schemes. The 0 and −1 frame products are labeled as indicated. (C) Relative frameshifting activity of (B) with the frameshifting efficiency of construct 13390–13520 as 100% (for comparison purposes). Value for each construct was the mean of three independent experiments, with the bar representing the standard error of the mean.

To search for a minimal determinant within ATT, we performed sequential 5′sequence deletions of ATT and compared the relative frameshifting activity of the different deletion variants (Fig. 1). We found that viral sequences, covering nucleotides 13363 to 13387, possessed substantial−1 PRF attenuation activity (Fig. 1) and the ability to form a stable stem-loop structure (Fig. 2A). However, the base of the predicted hairpin stem is only 4 nucleotides away from the 5′-edge of the slippery site. It is possible that a hairpin stem cannot be formed when the slippery site occupies ribosomal P and A sites.

**Figure 3:**
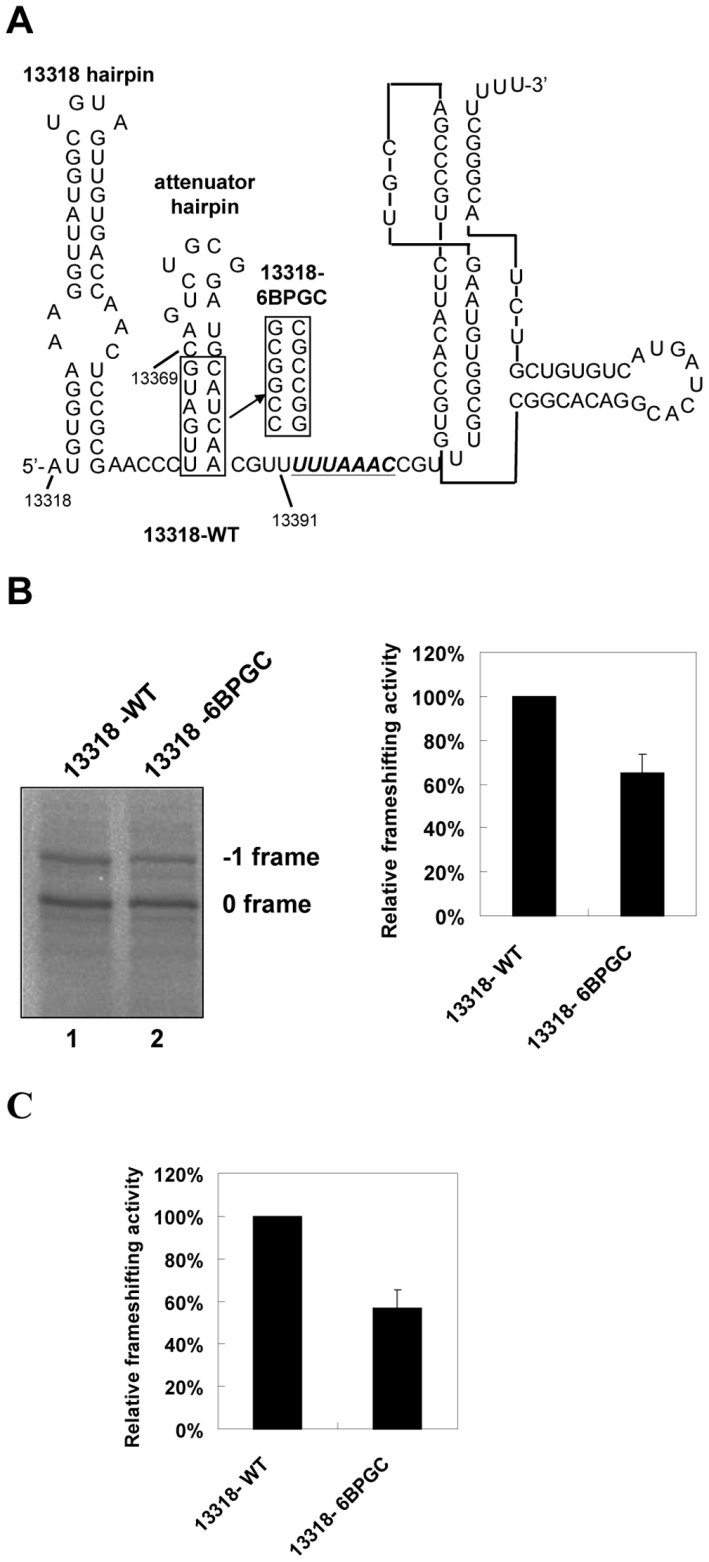
Sequence composition of the predicted hairpin stem is crucial for its −1 PRF attenuation activity. (A) The scheme for swapping the base-pairing composition of the attenuation hairpin stem in constructs containing a longer SARS-CoV viral sequence. The covered viral sequences (13318 to 13391) and the predicted secondary structure are shown, with the six swapped base pairs boxed for comparison, while the slippery site is underlined and followed by a SARS-CoV pseudoknot (SARS-PK) stimulator. (B) *In vitro* −1 PRF assays by SDS-PAGE analysis of ^35^S methionine-labeled translation products for constructs in (A) (left), and the relative frameshifting activity with that of 13318-WT being treated as 100% (right). Error bars, s.d.; *n* = 3. (C) Relative frameshifting activity calculated from dual-luciferase assay data using 293T cells harboring transiently expressed 13318-WT and 13318-6BPGC constructs with the frameshifting efficiency of 13318-WT being treated as 100%. Error bars, s.d.; *n* = 3.

In the past, the refolding pathway of unwound RNA structures within ribosome cores has not been comprehensively investigated. Interestingly, the crystal structure of an elongation mode of the 70S ribosome indicates that nucleotides within the first codon upstream of the E codon are flexible [17]. The implication being that these nucleotides are accessible for base-pair formation. Therefore, we disrupted two potential AU base pairs in the lower stem of the predicted hairpin to generate two AC mismatched mutations at the 13363–13520 construct. We found that the resultant 5′CC-WT construct lost two-thirds of its attenuation activity compared with an intact hairpin (Figs. 2B and 2C). Because both 5′CC-WT and 13363–13520 constructs share 27 identical nucleotides upstream of their slippery sites, the attenuation activity difference is not likely to be caused by an E-site flanking sequences effect [12], [13] but rather by the disruption of the two potential AU base pairs. Similar results were observed for GC-SB-WT and 6BPGC hairpins when potential Watson-Crick base pairs were disrupted (Figs. 2B and 2C). Together, these results suggest that base-pair formation and the composition of the predicted hairpin stem are crucial for efficient attenuation. Next, we swapped six GC base pairs for six corresponding wild-type base pairs in the predicted hairpin stem within a longer SARS-CoV viral sequence (13318-WT) (Fig. 3A) and found that the attenuation activity of the chimera was further enhanced both *in vitro* and in 293T cell cultures (Fig. 3B and 3C), indicating that the predicted stem-loop is a major determinant of −1 PRF attenuation activity in SARS-CoV ATT.

### Potential Base Pairs Involving E Site Sequences are not Essential for Attenuation

We noticed a potential to form four extra base pairs between 5′- and 3′- flanking sequences (GACG and CGUU, respectively) of the 6BPGC hairpin stem (and other deletion mutants) due to the existence of a 5′ *Sal*I cloning site (Fig. S1A). In particular, the formation of two base pairs involving 3′- flanking UU invaded the 0 frame E site of the ribosome when the slippery sequence occupied ribosomal P and A sites. This invasion could interfere with the proposed reading-frame maintenance function of the E site [18]. However, mutagenesis analysis indicates that *in vitro* attenuation activity of the 6BPGC hairpin is not sensitive to base-pair formation involving 3′- flanking UU sequences (Figs. S1A and S1B). On the other hand, disrupting potential base pairs at 3′- flanking CG impaired attenuation activity by a third (Fig. S1B compares UUCG-6BPGC with GAAA-6BPGC). Especially important is the disruption of two GC base pairs at the bottom of the hairpin stem (6BPGC12AG). Such a disruption dramatically reduced attenuation activity. This evidence seems to show that two extra GC base pairs involving 3′- flanking CG lead to an extended stem and contribute to attenuation activity. However, only one potential GC base pair exists in the corresponding region of wild-type SARS-CoV viral RNA sequences (Fig. 3A), suggesting that this extra base pair is not essential for attenuation activity by the wild-type SARS-CoV attenuator hairpin. To see if these observations are valid and applicable to a different stimulator in other biological systems, selected 6BPGC 5′-flanking sequence mutants were placed upstream of a distinct −1 PRF stimulator, the DU177 pseudoknot [19], and examined for their attenuation activity in 293T cells. The results (Fig. S1C) indicate that the two potential base pairs involving E-site sequences are not the main cause of observed attenuation activity in 293T cell cultures.

### Attenuation Efficiency is Positively Correlated with Hairpin Stability

**Figure 4:**
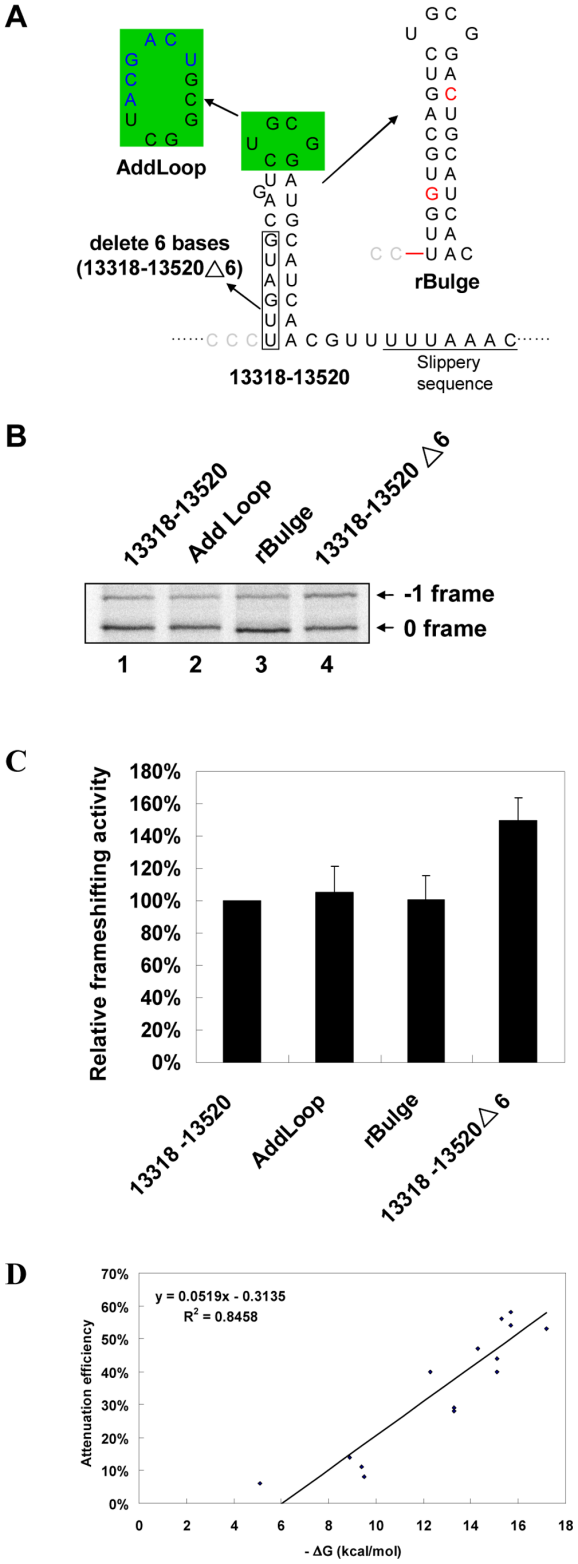
The UGCG loop and G bulge are not the major determinants for −1 PRF attenuation whereas the attenuation efficiency of a hairpin is positively correlated with its stability. (A) Illustration of constructs with G-bulge or UGCG loop (shaded in green) disruption and with 5′-half sequence deletion (boxed) of the lower stem of the predicted hairpin. The bulge G was converted into a GC base pair by inserting a C in its complementary strand. An upstream C nucleotide was deleted and accompanied with an A to G replacement to correct the frame and prevent an in-frame stop codon, respectively. The modified sequences in AddLoop and rBulge constructs are typed in blue and red, respectively. (B) *In vitro* −1 PRF assays by SDS-PAGE analysis of ^35^S methionine-labeled translation products for constructs in (A). The 0 and −1 frame products are labeled as indicated. (C) Relative frameshifting activity of (B). Frameshifting efficiency of the construct 13318–13520 was treated as 100% for comparison. Value for each construct was the mean of three independent experiments, with the bar representing the standard error of the mean. (D) Correlation between attenuation efficiencies (against the 13390–13520 construct) and predicted free energy values of the 6GC-hairpin variants in Fig. S2. A linear regression line is shown with the equation for the line and the regression statistic, R^2^, and a threshold stability below which the hairpin does not attenuate frameshifting. Attenuation efficiency was calculated according to the definition given in experimental procedures.

The identified attenuator hairpin contained a single nucleotide G bulge and a UGCG tetra-loop in the upper part of its stem. We examined the roles of both motifs in attenuation; however, neither insertion of a C nucleotide to convert the G bulge into a GC pairing nor the six nucleotides inserted to interrupt UGCG loop sequences impaired attenuation efficiency significantly (Figs. 4A to 4C). By contrast, deletion of 6 nucleotides at the 5′-half of the lower stem in the wild-type SARS CoV 13318–13520 construct abolished attenuation activity (Figs. 4A to 4C). Thus, the apical UGCG loop and G bulge are not major determinants of attenuation.

Next, we investigated the role of stability in the potency of attenuator hairpins by designing a simplified RNA hairpin with only 6 GC base pairs (6GC-hairpin). We found that it possessed attenuation activity comparable to that of a 6BPGC hairpin (Figs. S2A and S2 B). We further modified the composition of base-pairings along the hairpin stem to create variants of different attenuation activity (Figs. S2A and S2B). A plot of attenuation efficiencies against predicted free energy values for these 6GC-hairpin variants reveals a positive correlation between both parameters (Fig. 4D), indicating that hairpin stability is crucial for attenuation efficiency.

### Spacing to the Slippery Site Determines Whether a Hairpin Acts as an Efficient −1 PRF Attenuator

**Figure 5:**
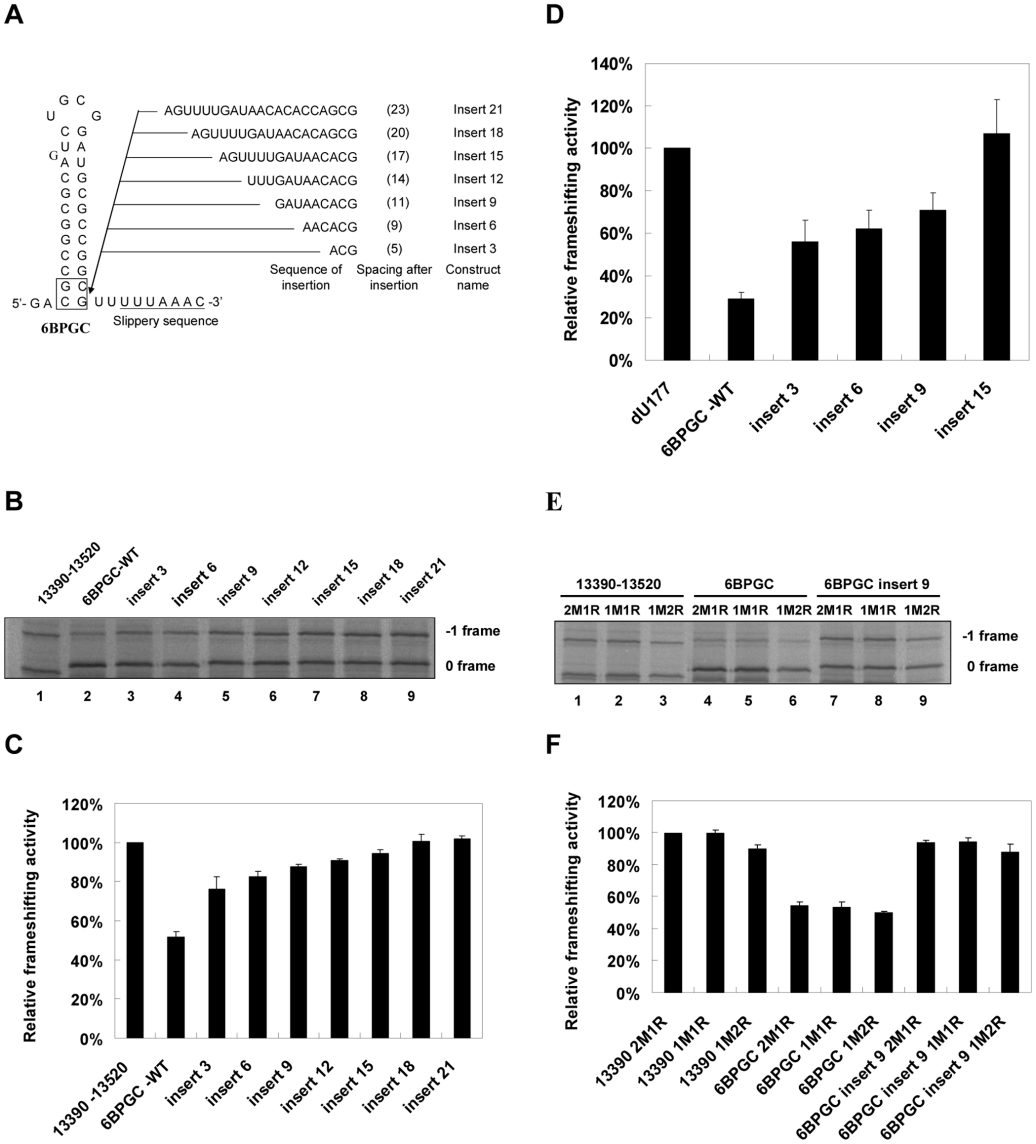
Proximity to the slippery site determines the attenuation potency of a hairpin. (A) Scheme showing the inserted nucleotide sequences between the extended base of the 6BPGC hairpin stem and the slippery site with spacing numbers after insertion shown in parentheses. The two extended base pairs involving spacer are boxed. The inserted sequences were designed to retain no stable secondary structure and regenerate the 3′-flanking CGUU sequence to prevent flanking sequence effects affecting frameshifting efficiency [12], [13]. (B) *In vitro* −1 PRF assays by SDS-PAGE analysis of ^35^S methionine-labeled translation products for constructs with insertions as shown in (A). (C) Relative frameshifting activity with that of the construct 13390–13520 being treated as 100%. Error bars, s.d.; *n* = 3. (D) Relative −1 frameshifting activity based on dual-luciferase assays from yeast cells, transfected with reporters containing selected insertion mutants in (A) and SARS-PK replaced by DU177 pseudoknot. Frameshifting efficiency of the construct containing the DU177 pseudoknot alone was treated as 100% (for comparison purposes). Error bars, s.d.; *n* = 3. (E) *In vitro* −1 PRF assays by SDS-PAGE analysis for reporter constructs (with or without nucleotide insertion in the region between the extended base of 6BPGC hairpin stem and the slippery site) under different conditions with variations in relative amounts of mRNA and Retic lysate. Condition designations: 2M1R (mRNA 100 ng/Retic lysate 1.7 μl); 1M1R (mRNA 50 ng/Retic lysate 1.7 μl); 1M2R (mRNA 50 ng/Retic lysate 3.4 μl) in a total of 5 μl/reaction. (F) Relative frameshifting activity of (E) using frameshifting efficiency of 13390–13520 construct in 2M1R condition as 100%. Error bars, s.d.; *n* = 3.

In addition to reducing hairpin stability, base-pairing disruption at the lower stem leads to changes in the spacing between the bottom of the hairpin stem and the slippery site. To address this issue, we created mutants by inserting different numbers of nucleotides between the two extra GC base pairs of the extended 6BPGC hairpin stem and the slippery site (Fig. 5A). When the spacing was increased from 2 to 5 nucleotides, attenuation activity was reduced by about a half. It was reduced further by the insertion of additional nucleotides (Figs. 5B and 5C). The spacing dependency of attenuation activity in yeast and a construct containing the DU177 pseudoknot stimulator was also examined and confirmed (Fig. 5D). Varying the ratio between mRNA and ribosomes in *in vitro* assays so that the amount of ribosomes available for each mRNA was different did not affect attenuation activity significantly (Figs. 5E and 5F). This result indicates that the lower attenuation activity attributable to a distant hairpin is not likely due to hairpin unwinding by an adjacent marching ribosome. Cumulatively, these results establish the following: 1) attenuation activity of a hairpin depends on its spacing to the slippery site; 2) attenuation functions are preserved among distinct eukaryotic systems; and 3) attenuators down-regulate distinct −1 PRF stimulators.

### Attenuation Activity is not Sensitive to E Site Sequence Variation

**Figure 6:**
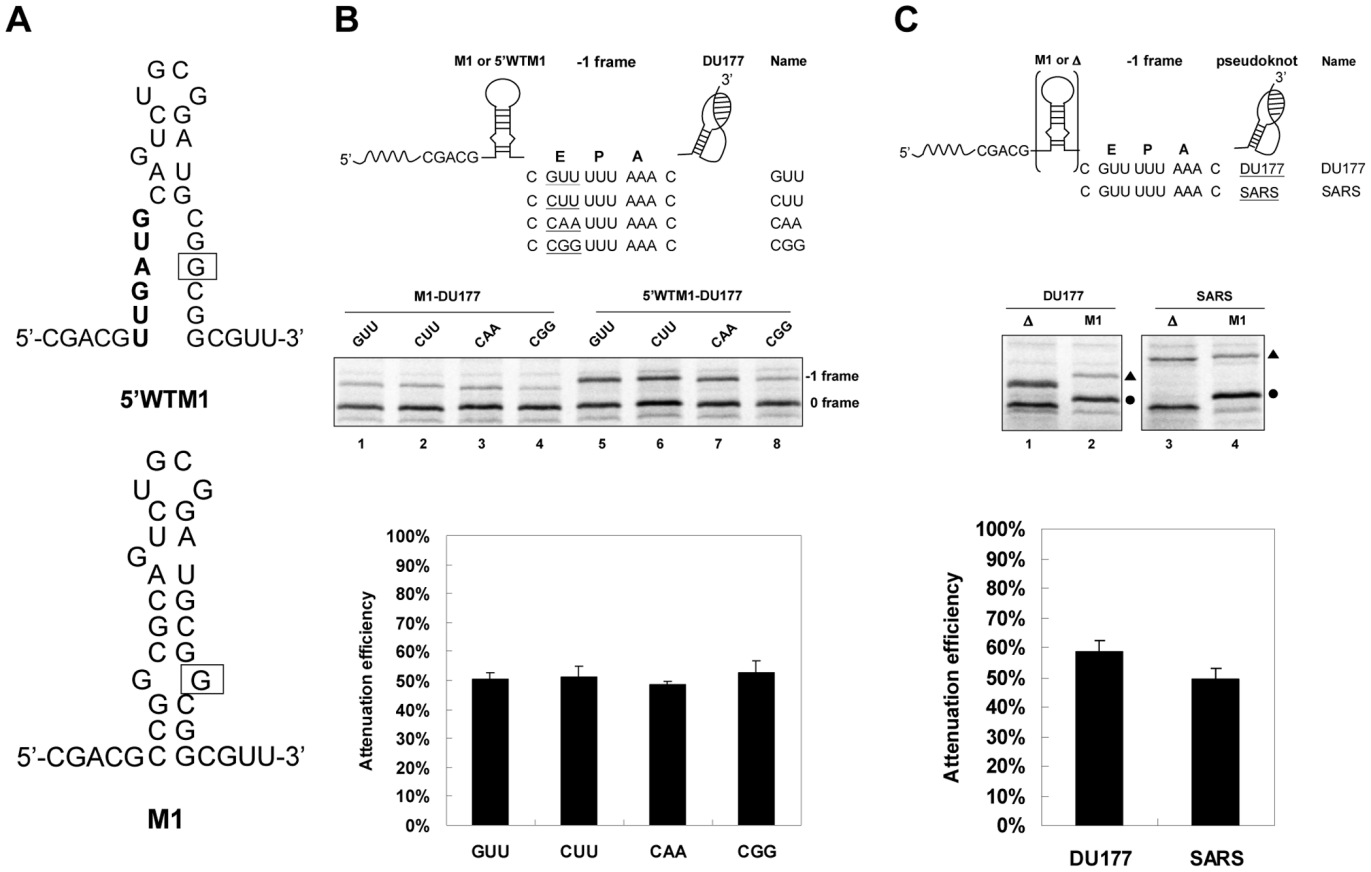
Effects of E Site sequences and downstream pseudoknot stimulator identities on the attenuation activity of M1 hairpin. (A) The sequences of M1 attenuator hairpin with the mutation site boxed and 5′WT-M1 element with the mutation sites boldly typed.(B) The sequence variation (top), SDS PAGE result of −1 PRF assays (middle) and attenuation efficiencies of M1 attenuator (bottom) of reporter constructs with E site sequence variation. Error bars, s.d.; *n* = 3. (C) The −1 PRF module set-up (top), SDS PAGE result of −1 PRF assays (middle), and attenuation efficiencies of M1 attenuator (bottom) of reporter constructs with different downstream pseudoknot stimulators. Δ means the attenuator hairpin is deleted. The 0 frame and −1 frame products are designated by filled circles and triangles, respectively. Error bars, s.d.; *n* = 3.

In contrast to a downstream −1 PRF stimulator promoting −1 frameshifting, a potential hairpin attenuator upstream of the slippery site possesses an opposing effect. As E site sequences can affect the potency of downstream stimulators [12], [13], we ask whether the potency of attenuators can be affected by either proximal E site sequences or downstream stimulators. A weakened M1 attenuator hairpin, derived from a 6BPGC hairpin with a disrupted GC base pair (in the middle of the hairpin stem (Fig. 6A)), and a potent DU177 −1 PRF stimulator were used to address these issues. They were chosen because a potent 6BPGC hairpin attenuated a weaker SARS −1 PRF stimulator so efficiently that the resultant intensity of radioactivity in *in vitro* assays gave uncertain results (data not shown). Consistent with flanking sequences effects, changing sequences in the −1 frame E site led to variations in −1 frameshifting efficiencies. These were promoted by the same stimulator regardless of the absence or presence of an M1 attenuator. However, calculated −1 PRF attenuation efficiencies of the M1 attenuator remained virtually unchanged among different E-site sequence variants (Fig. 6B), indicating that the attenuator down-regulates −1 PRF to a similar extent under flanking-sequences effects. By contrast, attenuation efficiencies of the M1 attenuator were different among constructs containing DU177 or SARS −1 PRF stimulator (Fig. 6C), implying discrimination toward distinct stimulators.

We considered the possible mechanisms by which an attenuator may interact with downstream stimulator or sequester soluble factors involved in −1 frameshifting stimulation. However, no obvious variation in attenuation activity was observed in −1 PRF reporters (with or without the *in-cis* attenuator hairpin) in the presence of different dosages of *in-trans* RNA attenuator hairpins (Fig. S3). This suggests that −1 PRF attenuation is not mediated by either mechanism. We then investigated the possibility that an attenuator could actively alleviate the proposed strain triggered by ribosomal helicase resistance to a −1 PRF stimulator [2], [8] and thus offset −1 frameshifting. Should this prove to be true, we predict that an efficient −1 PRF attenuator could facilitate frameshifting in the +1 direction under appropriate circumstances.

### The 6BPGC Hairpin Stimulates +1 Frameshifting in Yeast

**Figure 7:**
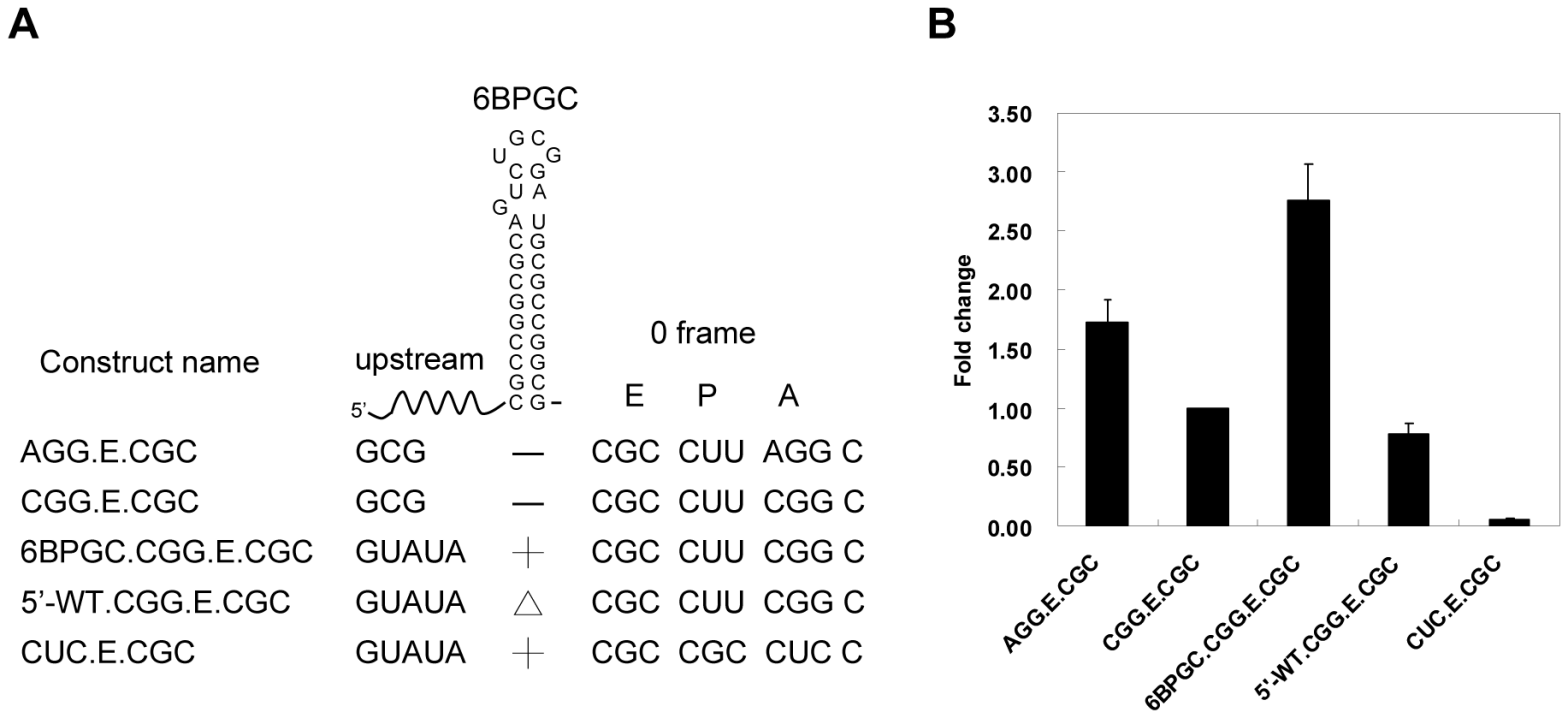
The 6BPGC hairpin serves as a +1 PRF stimulator when placed upstream of a +1 shifty site in yeast. (A) Schematic drawing for the flanking sequences surrounding the 6BPGC hairpin in yeast +1 frameshifting reporter constructs. Construct with a disrupted attenuator hairpin (5′-WT) was designated by Δ and the 5′-flanking sequence of 6BPGC hairpin was designed to prevent forming base-pairs with E site sequences. (B) Fold change of the AGG- and 6BPGC-dependent +1 frameshifting activity in yeast using the activity of the CGG.E.CGC construct as 1. Error bars, s.d.; *n* = 6.

A hepta-nucleotide sequence (CUUAGGC), derived from Ty1 retrotransposon of yeast *Saccharomyces cerevisiae*, can efficiently induce +1 PRF. It is caused by a ribosomal pause at the AGG codon due to the low expression levels of decoding tRNA in yeast. Mutating AGG to CGG partially impairs +1 frameshifting activity [21]. We reasoned that a partially functional CUUCGGC sequence represented an ideal platform for evaluating the properties of an upstream 6BPGC hairpin (Fig. 7). Indeed, a reporter construct of AGG-containing hepta-nucleotides possessed high +1 frameshifting activity (compared with random-sequence negative controls) whereas the +1 frameshifting activity of a reporter with CGG-containing hepta-nucleotides decreased significantly (Fig. 7). In this study, the smaller difference in +1 frameshifting efficiency between AGG and CGG constructs compared with that of Ty1 retrotransposon may result from a difference in E site sequence identity (CGC versus CAC) [22]. Importantly, we found that an upstream 6BPGC hairpin with 5′-flanking sequences designed to prevent direct E site invasion stimulated +1 frameshifting of the CGG-containing shift site (Fig. 7). By contrast, mutants carrying mutations to disrupt base pairs at the hairpin stem (5′-WT construct of Fig. 7) lost the ability to stimulate +1 frameshifting. Therefore, this upstream 6BPGC hairpin may act as a +1 frameshifting stimulator and is indeed a programmed reading-frame switch regulator.

## Discussion

In addition to affecting hairpin stability, mutations that change nucleotide composition in a hairpin stem can alter the encoded amino acids. To see if the nature of amino acids encoded by a hairpin is responsible for the observed variation in −1 PRF attenuation, mutation data and the amino acids encoded in particular attenuator hairpin variants in this study were further analyzed (Table S1). Comparison of the encoded amino acids between constructs 13363–13520 and 5′CC-WT (Fig. 2) in mutation sites that disrupts 2 AU base pairs reveals a leucine to proline change. By contrast, a similar leucine to proline change from constructs 3′GG-WT to GC-SB-WT (Fig.2) increased −1 PRF attenuation activity. Thus, reduced −1 PRF attenuation activity in 5′CC-WT is not caused by the replacement of encoded leucine with proline in the mutation sites. Additionally, amino acid compositions encoded by 6BPGC, r-Bulge, and 6GC hairpin (Fig. 4 and Fig. S2) are changed further (Table S1) with the maintenance of substantial −1 PRF attenuation activity. Together, these observations suggest that changes in the encoded amino acid composition caused by nucleotide mutations at the predicted hairpin stems are not a major determinant of −1 PRF attenuation.

Refolding of a ribosomal unwound RNA structure within a ribosome has not been fully addressed; however, a previous study indicates that a length of about 30 nucleotides is protected from ribonuclease digestion when mRNA is occupied by a prokaryotic ribosome [23]. Our observation of a positive correlation between hairpin stability and attenuation efficiency (Fig. 4D) strongly suggests that base-pair reformation of the upstream hairpin stem plays a crucial role in the reduction of −1 PRF efficiency. Furthermore, mutating two nucleotides (27 nucleotides upstream of the E site) to disrupt Watson-Crick base pairs in the lower hairpin stem dramatically impairs attenuation activity (Fig. 2), indicating that attenuation is not caused by primary sequence-mediated flanking-sequences effects [12], [13]. Together, these observations support the idea that a ribosomal unwound hairpin stem can partially reform when the final codon in the 3′-half of the lower stem leaves the E site. The proposed ribosomal fall-off hypothesis that has original ATT impeding ribosome processivity [15] could work here. However, such a mechanism should decrease the observed efficiencies for both +1 PRF and −1 PRF. Additionally, the 6GC hairpin used in this work is not likely to cause a ribosome to fall off because the ribosomal helicase is capable of unwinding a duplex of 27 base pairs [1]. This raises the question of what the other potential mechanisms responsible for programmed reading-frame regulation by a refolding hairpin are.

Although final proof of the existence of a refolding hairpin stem proximal to the ribosomal E site during −1 PRF stimulation awaits direct physical evidence such as ribosome crystallography and mRNA : rRNA cross-linking analysis, the available 70S ribosome structure mimicking the elongation stage of translation indicates that duplex formation between SD and anti-SD can exist 5 to 6 nucleotides upstream of the P site [17] and overlaps the region where the cis-acting hairpin stem reforms. Additionally, Cryo-EM structures of the 80S ribosome-bound viral internal ribosomal entry site indicate that a folded structure can be accommodated in the space surrounding the mRNA exit site [24], [25]. In this context, SD⋅anti-SD duplex formation has been shown to change the mRNA exit channel pathway and create numerous ribosomal interactions [26]. Thus, formation of a refolding cis-acting hairpin stem could create contacts with the 80S ribosome to modulate the E site network and regulate a programmed reading-frame switch. In a non-mutually exclusive model, the +1 frameshifting stimulation and −1 PRF attenuation properties of a refolding hairpin can be explained by a pulling force in the 5′-direction generated by hairpin stem closure. This explanation is consistent with proposed mechanical tension triggered by a −1 PRF stimulator [2]. In agreement with this active role, a refolding GC-rich hairpin has been shown to exert a 5′-pulling force on RNA-DNA hybrids at the active site of RNA polymerase [27]. Alternatively, the upstream hairpin may serve as a wheel chock that blocks −1 ribosomal movement during late stage of hairpin refolding. Thus, a stable hairpin upstream of the slippery site represents a cis-acting RNA motif for −1 PRF attenuation. This is in contrast to the downstream −1 PRF stimulator. These revelations should be of much interest when further studies on the programmed frameshifting mechanism are being planned. By contrast, a much more stable structure, such as the original ATT in SARS CoV, could still affect viral −1 PRF efficiency by serving as a translational attenuator as previously proposed [15]. However, deletion of six nucleotides to disrupt the minimal upstream hairpin stem in an ATT-containing construct (construct 13318–13520 Δ6 in Fig. 4B) restored −1 PRF efficiency to that of the ATT-lacking 13390–13520 construct *in vitro* (compare lanes 1 and 4 of Fig. 4B and Fig. 1B).

Interestingly, both the *in-cis* acting hairpin revealed here and the internal SD⋅anti-SD interaction in 70S ribosome can stimulate +1 PRF as well as attenuate −1 PRF when placed in close proximity upstream of the corresponding shift sites [11], [18]. However, moving the cis-acting attenuator hairpin 5′ further reduced its −1 frameshifting attenuation activity (Fig. 5), whereas moving the internal SD⋅anti-SD duplex further upstream of the slippery site converted the duplex into a −1 PRF stimulator [11]. A possible reason is that the 16S rRNA component of 70S ribosome is part of the functional duplex, whereas the eukaryotic ribosome does not have an anti-SD sequence. It will be interesting to see if an in-cis acting RNA hairpin can replace the functionality of internal SD⋅anti-SD interaction in the 70S ribosome.

Further experiments, such as measuring stimulator unwinding and attenuator hairpin refolding times (by the single-molecule approach) [2] as well as elucidating how translational machinery responds to a refolding hairpin should help reveal the interplay responsible for the intricacies of reading-frame switch adjustment. Finally, the search for overlooked cis-acting regulators in the programmed reading-frame switches of genomes using bioinformatics should benefit from the stability and proximity features revealed in this study. Because widely distributed RNA structures along an open reading-frame [28] are unwound and refolded repeatedly during translation, the involvement of refolding RNA hairpins in the regulation of translational elongation may be more common than first thought.

## Materials and Methods

### Plasmids

The plasmid encoding the gene for ORF 1ab junction region of SARS-CoV, pCRII-SARS_12265–13653_ was a gift from Professor Pei-Jer Chen at National Taiwan University. The p2luc recoding reporter [29] suitable for both +1 and −1 frameshifting assays was a kind gift from Professor John Atkins at the University of Utah. Plasmids pJD-375 & 378 [30] were obtained from Professor Jonathan Dinman at the University of Maryland, while the yeast strain yRP1674 [31] was a gift from Professor Roy Parker at the University of Arizona.

### Construction of Reporter Genes and Mutagenesis

Forward and reverse DNA primers, respectively carrying the *Sal*I and *BamH*I restriction sites and appropriately designed annealing sequences, were used for PCR amplification of the desired cDNAs encoding SARS-CoV viral RNAs by using pCRII-SARS_12265–13653_ as the template. The pseudoknot stimulator sequences of DU177 [19] with or without the 6BPGC attenuator hairpin were chemically synthesized. The amplified inserts of interests were then cloned into the *Sal*I/*BamH*I sites of p2luc using standard procedures and the resultant recombinant vectors were transformed into DH5α strain of *E. coli* cells for maintenance and selection by ampicillin. All of the base-pairing disruption and restoration mutants were constructed using a quik-change mutagenesis kit from Stratagene according to the manufacturer’s instructions. For cloning reporter constructs suitable for *in vivo* frameshifting assays in yeast, inserts of interest were treated as above and cloned into *Sal*I/*BamH*I restriction sites of the pYDL-empty reporter (see below). Identities of all cloned and mutated genes were confirmed by DNA sequence analysis.

As the original pJD378 plasmid possesses an inserted HIV recoding signal between *BamH*I/*Sac*I restriction sites, an insert-free vector, derived from pJD378, was created to facilitate subsequent cloning of recoding signals of interests. To this end, the gene fragment corresponding to the HIV recoding signal and ensuing firefly luciferase ORF in pJD378 were removed by treatment with restriction enzymes, *BamH*I and *Xho*I. An insert-free region corresponding to that in p2Luc plasmid was obtained by PCR amplification using a set of forward and reverse primers containing *BamH*I and *Xho*I recognition sequences, respectively. The amplified inserts were treated by the same set of restriction enzymes after purification. Both fragments were then purified, recovered and ligated to obtain a recombinant insert-free pJDL-empty vector. This pJDL-empty vector has the same set of cloning sites as those in p2Luc, making it suitable for insertion with other recoding signals. Identities of all cloned and mutated genes were confirmed by DNA sequencing analysis.

### DNA/RNA Synthesis and Purification

Synthetic DNA oligonucleotides used in this study were chemically synthesized and purchased from MISSION BIOTECH**.** Synthetic RNAs used in this study were transcribed by T7 RNA polymerase with designed DNA templates using *in vitro* transcription methods [32] **.** After being purified by 20% denaturing polyacrylamide gel electrophoresis in the presence of 8 M urea, gels of bands containing RNA of desirable sequences were cut out and electro-eluted using a BIOTRAP device (Schleicher & Schuell). The eluted RNAs were then ethanol precipitated and recovered by centrifugation. Finally the concentration of a particular DNA or RNA was determined by UV absorbance at 260 nm.

### Radioactivity-based *in vitro* −1 PRF Assay

Capped reporter mRNAs were prepared using the mMESSAGE mMACHINE high-yield capped RNA transcription kit (Ambion) following the manufacturer’s instructions. Reticulocyte lysate (Progema or Ambion) was used to generate shifted and non-shifted protein products. In each assay, a reaction totaling 5 μl of reactants (i.e., 50–250 ng of capped reporter mRNA, 2.5 μl of reticulocyte lysate, and 0.2 μl of 10 μCi/μl ^35^S-labeled methionine (NEN)) was incubated at 30°C for 1.5–2 hours. Samples were then resolved by 12% sodium dodecyl sulfate polyacrylamide gel electrophoresis (SDS-PAGE), and exposed to a phosphorImager screen for quantification on BAS-2500 phosphorImager (Fujifilm) or Typhoon FLA7000 phosphorImager (GE) after drying.

### Measurement of Attenuation Efficiency as Well as Application of Shortened −1 Frame Translation Products in Radioactivity-based -1 PRF Analysis *in vitro*

To facilitate −1 PRF activity analysis *in vitro*, the SARS-CoV 13222–13520 fragment was originally cloned into SalI/BamHI sites of p2luc so that shifted ribosomes would encounter a premature −1 frame stop codon, located 33 nucleotides downstream of the corresponding BamHI site, and produce a shortened −1 frame product during translation [19]. All the other 5′-deletion mutants derived from the SARS 13222–13520 construct in Fig. 1 (including the 13363–13520 construct) possess this property. All radioactivity-based −1 PRF activity measurement *in vitro* was performed assuming that the ribosome drop-off effect [29] was minimized for the translation of a shortened −1 frame product. As we present most of our *in vitro* −1 PRF results in terms of relative −1 PRF activity, ribosome drop-off effect is removed. Experiments were performed in triplicate and reported as one standard deviation from the mean.

Frameshifting efficiencies were calculated by dividing the counts of the shifted product by the sum of the counts for both shifted and non-shifted products. Calibration was conducted for the methionine content in each protein product. We also used relative frameshifting activity to compare attenuation activity among constructs with variations in attenuator composition, spacing to the slippery site, and E site sequence identity in the same gel. Attenuation efficiency of an upstream hairpin was defined as the difference in frameshifting efficiency between two constructs with or without an upstream hairpin, divided by the frameshifting efficiency value of the construct without the upstream hairpin. Experiments were performed in triplicate and reported as one standard deviation from the mean.

### Mammalian Cell Cultures and Frameshifting Assays

Human embryonic kidney HEK-293T cells were cultured in Dulbecco’s Modified Eagle Medium (Gibco) supplemented with 10% fetal bovine serum. One day before the transfection, 0.5–1×10^5^ HEK-293T cells per well were placed in a 24-well culture plate with 1000 μl growth medium. Transfection was conducted by adding the mixture of 0.5 μg plasmid DNA and jetPEI™ transfection reagent (Polyplus) into each well, according to the manufacturer’s instructions. Luciferase activity measurements for transfected 293T cell lysates were performed using the Dual Luciferase™ reporter assay (Promega) according to the manufacturer’s instructions on a CHAMELEON™ multi-label plate reader (HIDEX). All the experiments were repeated three times with four to six assays for each reaction. Frameshifting efficiency was then calculated according to previously described procedures [29]. The *firefly*/*Renilla* activity ratio generated from the control reporter was divided into that from frameshift reporters carrying frameshifting signals of interest and multiplied by 100 to obtain frameshifting efficiencies (expressed as percentages) for each recoding signal.

### Yeast Cell Cultures and Frameshifting Assays

To measure −1 and +1 PRF activity in yeast, yRP1674 cells (*Mat a his3*Δ*1 leu2*Δ *met15*Δ *ura3*Δ) [31] harboring pYDL-based reporter constructs were grown in liquid media composed of a minimal SD base with -Ura Do supplement (Clonetech) to an O.D._595 nm_ value of 1.0 on a 1 mL scale. Cells were harvested by centrifugation, washed once with 1 mL of ice cold lysis buffer (1× PBS pH 7.4, 1 mM PMSF) and then re-suspended in 0.3 mL of the same buffer. Cells suspensions were lysed with glass beads by agitation in a vortex mixer at 4°C for 3 minutes. Dual-luciferase activities were determined using 20 μL lysate/sample by a Dual-Luciferase Assay System (Promega) and on a CHAMELEON™ multi-label plate reader (HIDEX). Frameshift efficiencies were calculated using the method previously described [29], except that the pJD375 reporter [30] was used as a control to measure the *firefly*/*Renilla* activity ratio. All assays were performed in triplicate.

## Supporting Information

Figure S1 **Potential base pairs involving the E site sequences are not essential for attenuation.** (A) The 5′- flanking sequences GACG (typed in green) of 6BPGC hairpin are part of the *Sal*I restriction site (underlined) used during cloning, and have the potential to form base pairs with the 3′- flanking sequences CGUU (also typed in green) of the hairpin to generate four extra base pairs (connected by dashed lines) in the bottom of an attenuator hairpin stem. The 5′- flanking nucleotides mutated for disrupting potential base-pairings are listed below the drawing and typed in red with the number of potential base pairs left after disruption shown in parentheses. The 2 terminal GC base pairs disrupted in 6BPGC12AG for comparison are boxed. (B) *In vitro* -1 PRF assays by SDS-PAGE analysis of ^35^S methionine-labeled translation products for reporter constructs in (A) (left) and the relative frameshifting activity calculated by treating that of construct 13390–13520 as 100% (right). Error bars, s.d.; *n* = 3. (C) Relative frameshifting activity calculated from dual-luciferase assay data obtained from 293T cells harboring transiently expressed p2Luc reporters. The reporters contain 6BPGC 5′-flanking sequence mutants with the SARS-PK replaced by DU177 pseudoknot. The frameshifting efficiency of a reporter construct containing a disrupted 6BPGC hairpin attenuator (6BPGC5′WT-DU177) was used for comparison and treated as 100%. Error bars, s.d.; *n* = 3.
(TIF)

Figure S2 **Attenuation efficiency and predicted free energy of the 6GC-hairpin variants.** (A) The predicted secondary structures and free energy values (in kcal/mol) of all the 6GC-hairpin variants using Mfold [20]. Free energy prediction was performed using sequences that include the two extended GC base pairs involving spacer (boxed). The base pairs, which changed along the hairpin stem in each mutant, are typed in bold. All the variants share the same CGUU 3′-flanking sequence to minimize the E site flanking sequence effect. (B) *In vitro* -1 PRF assays by SDS-PAGE analysis of ^35^S methionine-labeled translation products for constructs containing variants of 6GC-hairpin of (A) above.
(TIF)

Figure S3 **The -1 PRF efficiency of a reporter with or without an** ***in-cis*** **potent attenuator is not affected by titration of an attenuator RNA** ***in-trans*** **.** (A) Schematic drawing of the reporter construct and the wild-type attenuator RNA hairpin used for *in-trans* titration. The SARS-PK was used as the stimulator in these -1 PRF reporters. (B) *In vitro* -1 PRF assays by SDS-PAGE analysis for the 6BPGC hairpin containing reporter in the presence of different amounts of *in-trans* WT attenuator hairpins (left), and the relative frameshifting activities in comparison with that of the reporter alone (right). The concentrations of the RNA hairpin are labeled as indicated. Error bars, s.d.; *n* = 3. (C) *In vitro* -1 PRF assays by SDS-PAGE analysis for attenuator-less reporter in the presence of different amounts of *in-trans* WT attenuator hairpins (left), and relative frameshifting activities in comparison with that of reporter alone (right). Error bars, s.d.; *n* = 3.
(TIF)

Table S1 **The nucleotide sequences and encoded amino acids of selected upstream -1 PRF attenuator hairpin variants.** The amino acids encoded by each 0-frame codon are shown below the codons, and the sequences corresponding to the 5′-half and 3′-half of each hairpin stem are boldly typed with the nucleotides involving particular base pairs disruption in two sets of constructs colored in red or blue.
(TIFF)

## References

[1] Takyar S, Hickerson R P, Noller HF (2005) mRNA helicase activity of the ribosome. Cell 120: 49–58. 15652481 10.1016/j.cell.2004.11.042

[2] Qu X, Wen JD, Lancaster L, Noller HF, Bustamante C, et al (2011) The ribosome uses two active mechanisms to unwind messenger RNA during translation. Nature 475: 118–121. 21734708 10.1038/nature10126 PMC4170678

[3] Stahl G, McCarty GP, Farabaugh PJ (2002) Ribosome structure: revesting the connection between translational accuracy and uncovential decoding. Trends Biochem. Sci. 27: 178–183. 10.1016/S0968-0004(02)02064-9 PMC7126812 11943544

[4] Buchan JR, Stansfield I (2007) Halting a cellular production line: responses to ribosomal pausing during translation. Biol. Cell 99: 475–487. 10.1042/BC20070037 17696878

[5] Farabaugh P J (1996) Programmed translational frameshifting. Microbiol. Rev. 60: 103–134. 10.1128/mr.60.1.103-134.1996 PMC239420 8852897

[6] Chamorro M, Parkin N, Varmus HE (1992) An RNA pseudoknot and an optimal heptameric site are required for highly efficient ribosomal frameshifting on a retroviral messenger RNA. Proc. Natl. Acad. Sci. USA 89: 713–717. 10.1073/pnas.89.2.713 PMC48309 1309954

[7] Giedroc DP, Cornish PV (2009) Frameshifting RNA pseudoknots: Structure and mechanism. Virus Res. 139: 193–208. 10.1016/j.virusres.2008.06.008 PMC2670756 18621088

[8] Plant EP, Muldoon Jacobs KL, Harger JW, Meskauskas A, Jacobs JL, et al (2003) The 9-Å solution: How mRNA pseudoknots promote efficient programmed -1 ribosomal frameshifting. RNA 9: 168–174. 12554858 10.1261/rna.2132503 PMC1237042

[9] Baranov PV, Gesteland RF, Atkins JF (2004) P-site tRNA is a crucial initiator of ribosomal frameshifting. RNA 10: 221–230. 14730021 10.1261/rna.5122604 PMC1370534

[10] Namy O, Moran SJ, Stuart DI, Gilbert RJC, Brierley I (2006) A mechanical explanation of RNA pseudoknot function in programmed ribosomal frameshifting. Nature 441: 244–247. 16688178 10.1038/nature04735 PMC7094908

[11] Larsen B, Wills NM, Gesteland RF, Atkins JF (1994) rRNA-mRNA base pairing stimulates a programmed -1 ribosomal frameshift. J. Bacteriol. 176: 6842–6851. 10.1128/jb.176.22.6842-6851.1994 PMC197052 7961443

[12] Kim YG, Maas S, Rich A (2001) Comparative mutational analysis of cis-acting RNA signals for translational frameshifting in HIV-1 and HTLV-2. Nucleic Acids Res. 29: 1125–1131. 10.1093/nar/29.5.1125 PMC29715 11222762

[13] Léger M, Dulude D, Steinberg SV, Brakier-Gingras L (2007) The three transfer RNAs occupying the A, P and E sites on the ribosome are involved in viral programmed -1 ribosomal frameshift. Nucleic Acids Res. 35: 5581–5592. 10.1093/nar/gkm578 PMC2018615 17704133

[14] Su MC, Chang CT, Chu CH, Tsai CH, Chang KY (2005) An atypical RNA pseudoknot stimulator and an upstream attenuation signal for -1 ribosomal frameshifting of SARS coronavirus. Nucleic Acids Res. 33: 4265–4275. 10.1093/nar/gki731 PMC1182165 16055920

[15] Plant EP, Rakauskaité R, Taylor DR, Dinman JD (2010) Achieving a golden mean: Mechanisms by which coronaviruses ensure synthesis of the correct stoichiometric ratios of viral proteins. J. Virol. 84: 4330–4340. 10.1128/JVI.02480-09 PMC2863758 20164235

[16] Hung M, Patel P, Davis S, Green SR (1998) Importance of ribosomal frameshifting for human immunodeficiency virus type I particle assembly and replication. J. Virol. 72: 4819–4824. 10.1128/jvi.72.6.4819-4824.1998 PMC110024 9573247

[17] Jenner LB, Demeshkina N, Yusupova G, Yusupov M (2010) Structural aspects of messenger RNA reading frame maintenance by the ribosome. Nat. Struct. Mol. Biol. 17: 555–560. 10.1038/nsmb.1790 20400952

[18] Márquez V, Wilson DN, Tate WP, Triana-Alonso F, Nierhaus KH (2004) Maintaining the ribosomal reading frame: The influence of the E site during translational regulation of release factor 2. Cell 118: 45–55. 15242643 10.1016/j.cell.2004.06.012

[19] Chou MY, Chang KY (2010) An intermolecular RNA triplex provides insight into structural determinants for the pseudoknot stimulator of -1 ribosomal frameshifting. Nucleic Acids Res. 38: 1676–1685. 10.1093/nar/gkp1107 PMC2836554 20007152

[20] Zuker M (2003) Mfold web server for nucleic acid folding and hybridization prediction. Nucleic Acids Res. 31: 3406–3415. 10.1093/nar/gkg595 PMC169194 12824337

[21] Belcourt MF, Farabaugh PJ (1990) Ribosomal frameshifting in the yeast retrotransposon Ty: tRNAs induce slippage on a 7 nucleotide minimal site. Cell 62: 339–352. 2164889 10.1016/0092-8674(90)90371-K PMC7133245

[22] Sanders CL, Curran JF (2007) Genetic analysis of the E site during RF2 programmed frameshifting. RNA 13: 1483–1491. 17660276 10.1261/rna.638707 PMC1950767

[23] Steitz JA (1969) Polypeptide chain initiation: nucleotide sequences of the three ribosomal binding sites in bacteriophage R17 RNA. Nature 224: 957–964. 5360547 10.1038/224957a0

[24] Spahn CMT, Jan E, Mulder A, Grassucci RA, Sarnow P, et al (2004) Cryo-EM visualization of a viral internal ribosome entry site bound to human ribosome: the IRES functions as an RNA-based translational factor. Cell 118: 465–475. 15315759 10.1016/j.cell.2004.08.001

[25] Schüler M, Connel SR, Lescoute A, Giesebrecht J, Dabrowski M, et al (2006) Structure of the ribosome-bound cricket paralysis virus IRES RNA. Nat. Struct. Mol. Biol. 13: 1092–1096. 10.1038/nsmb1177 17115051

[26] Yusupova GZ, Jenner L, Rees B, Moras D, Yusupov M (2006) Structural basis for messenger RNA movement on the ribosome. Nature 444: 391–394. 17051149 10.1038/nature05281

[27] Larson MH, Greenleaf WJ, Landick R, Block S (2008) Applied force reveals mechanistic and energetic details of transcription termination. Cell 132: 971–982. 18358810 10.1016/j.cell.2008.01.027 PMC2295211

[28] Kertesz M, Wan Y, Mazor E, Rinn JL, Nutter RC, et al (2010) Genome-wide measurement of RNA secondary structure in yeast. Nature 467: 103–107. 20811459 10.1038/nature09322 PMC3847670

[29] Grentzmann G, Ingram JA, Kelly PJ, Gesteland RF, Atkins JF (1998) A dual-luciferase reporter system for studying recoding signals. RNA 4: 479–486. 9630253 PMC1369633

[30] Harger JW, Dinman JD (2003) An in vivo dual-luciferase assay system for studying translational recoding in the yeast Saccharomyces cerevisiae. RNA 9: 1019–1024. 12869712 10.1261/rna.5930803 PMC1236998

[31] Passos DO, Doma MK, Shoemaker CJ, Muhlrad D, Green R, et al (2009) Analysis of Dom34 and its function in no-go decay. Mol. Biol. Cell 20: 3025–3032. 10.1091/mbc.E09-01-0028 PMC2704154 19420139

[32] Frugier M, Florentz C, Hosseini MW, Lehn JM, Giege R (1994) Synthetic polyamines stimulate *in vitro* transcription by T7 RNA polymerase. Nucleic Acids Res. 22: 2784–2790. 10.1093/nar/22.14.2784 PMC308248 8052534

