# Supplementary figures and images for: Regulation of Programmed Ribosomal Frameshifting by Co-Translational Refolding RNA Hairpins
Source: PLoS One. 2013 Apr 29;8(4):e62283. doi: 10.1371/journal.pone.0062283 (PMC3639245; doi:10.1371/journal.pone.0062283)

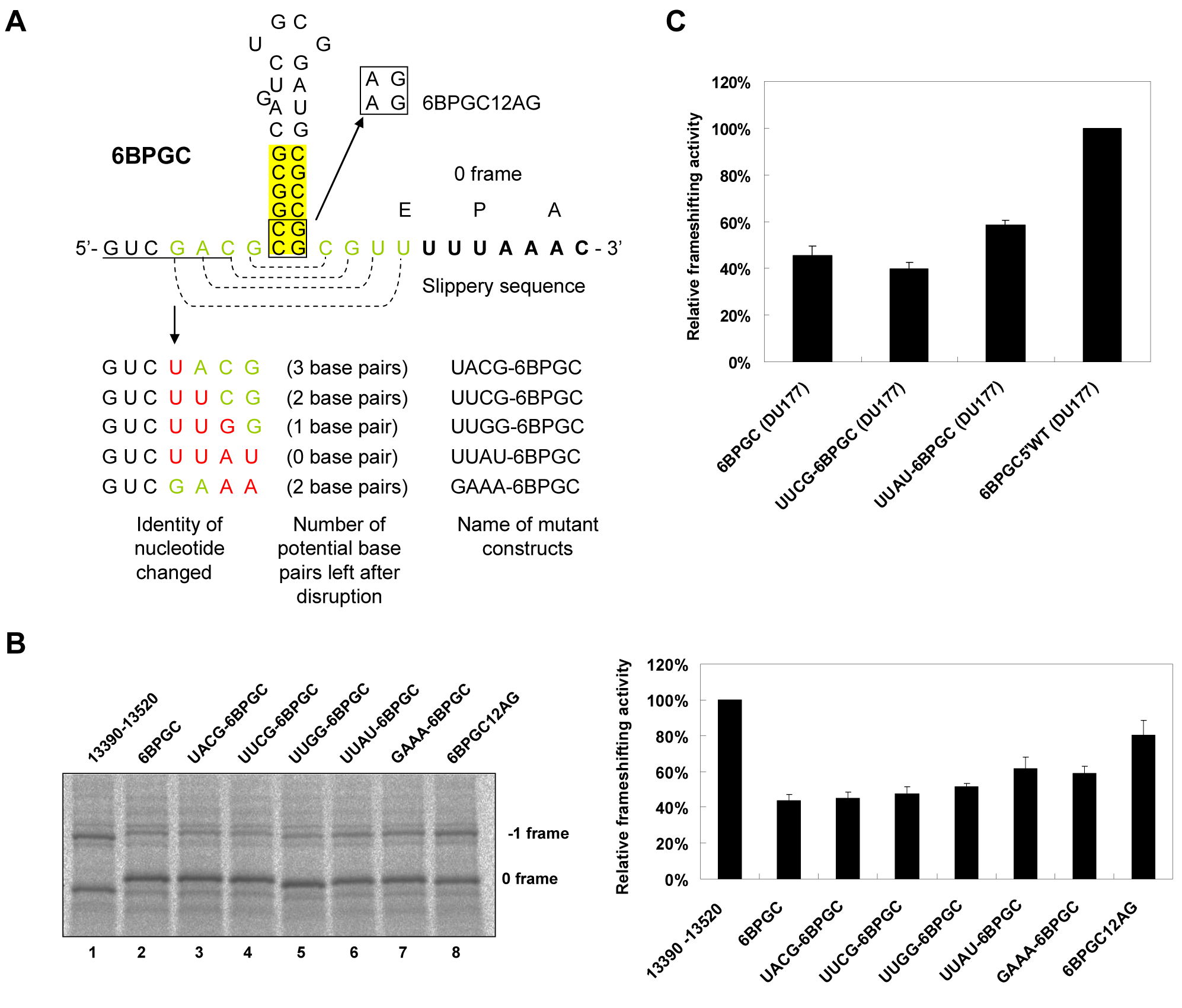

Supplement: Figure S1 — Potential base pairs involving the E site sequences are not essential for attenuation. (A) The 5′- flanking sequences GACG (typed in green) of 6BPGC hairpin are part of the SalI restriction site (underlined) used during cloning, and have the potential to form base pairs with the 3′- flanking sequences CGUU (also typed in green) of the hairpin to generate four extra base pairs (connected by dashed lines) in the bottom of an attenuator hairpin stem. The 5′- flanking nucleotides mutated for disrupting potential base-pairings are listed below the drawing and typed in red with the number of potential base pairs left after disruption shown in parentheses. The 2 terminal GC base pairs disrupted in 6BPGC12AG for comparison are boxed. (B) In vitro -1 PRF assays by SDS-PAGE analysis of 35S methionine-labeled translation products for reporter constructs in (A) (left) and the relative frameshifting activity calculated by treating that of construct 13390–13520 as 100% (right). Error bars, s.d.; n = 3. (C) Relative frameshifting activity calculated from dual-luciferase assay data obtained from 293T cells harboring transiently expressed p2Luc reporters. The reporters contain 6BPGC 5′-flanking sequence mutants with the SARS-PK replaced by DU177 pseudoknot. The frameshifting efficiency of a reporter construct containing a disrupted 6BPGC hairpin attenuator (6BPGC5′WT-DU177) was used for comparison and treated as 100%. Error bars, s.d.; n = 3. (TIF) [file pone.0062283.s001.tif]

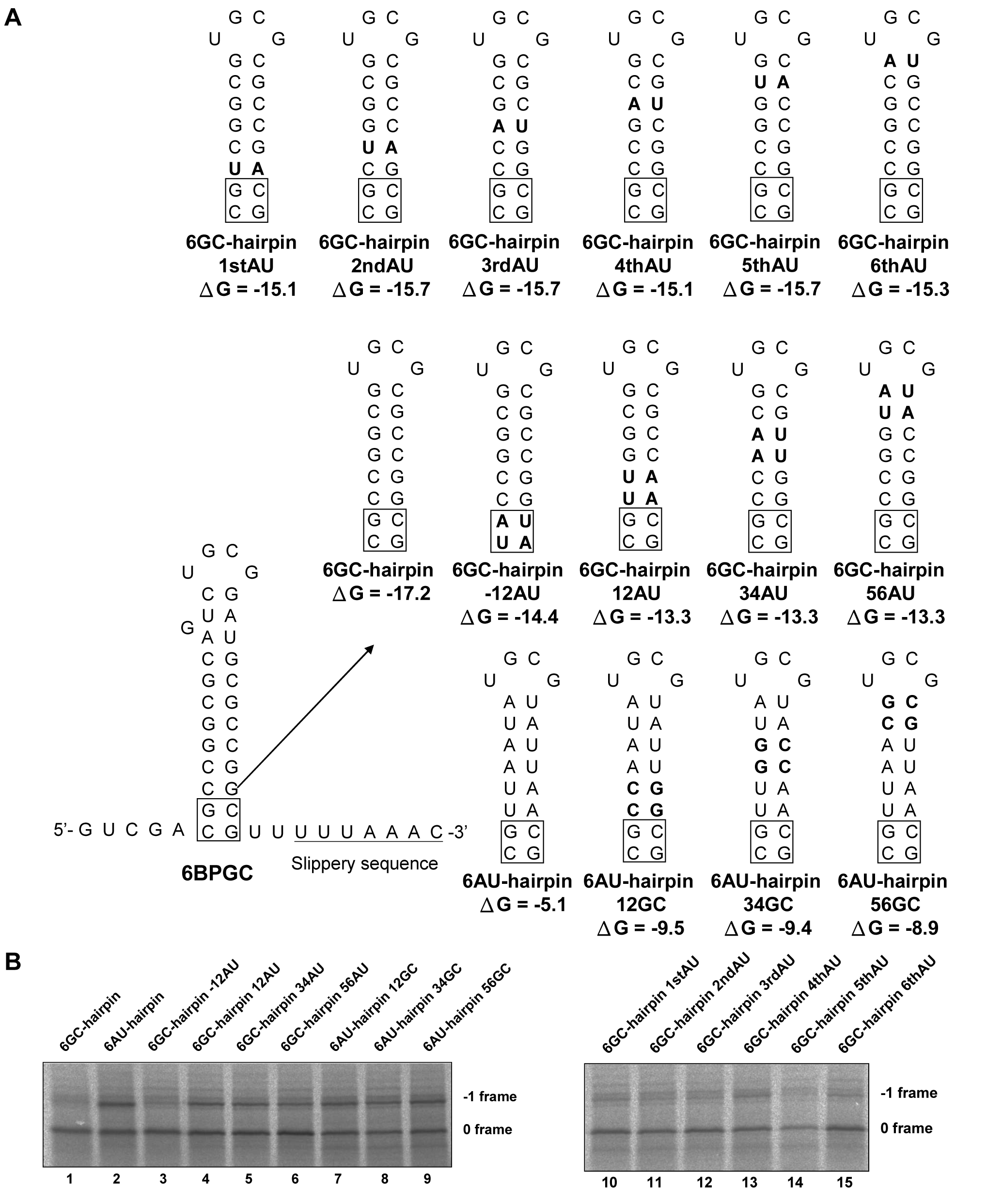

Supplement: Figure S2 — Attenuation efficiency and predicted free energy of the 6GC-hairpin variants. (A) The predicted secondary structures and free energy values (in kcal/mol) of all the 6GC-hairpin variants using Mfold [20]. Free energy prediction was performed using sequences that include the two extended GC base pairs involving spacer (boxed). The base pairs, which changed along the hairpin stem in each mutant, are typed in bold. All the variants share the same CGUU 3′-flanking sequence to minimize the E site flanking sequence effect. (B) In vitro -1 PRF assays by SDS-PAGE analysis of 35S methionine-labeled translation products for constructs containing variants of 6GC-hairpin of (A) above. (TIF) [file pone.0062283.s002.tif]

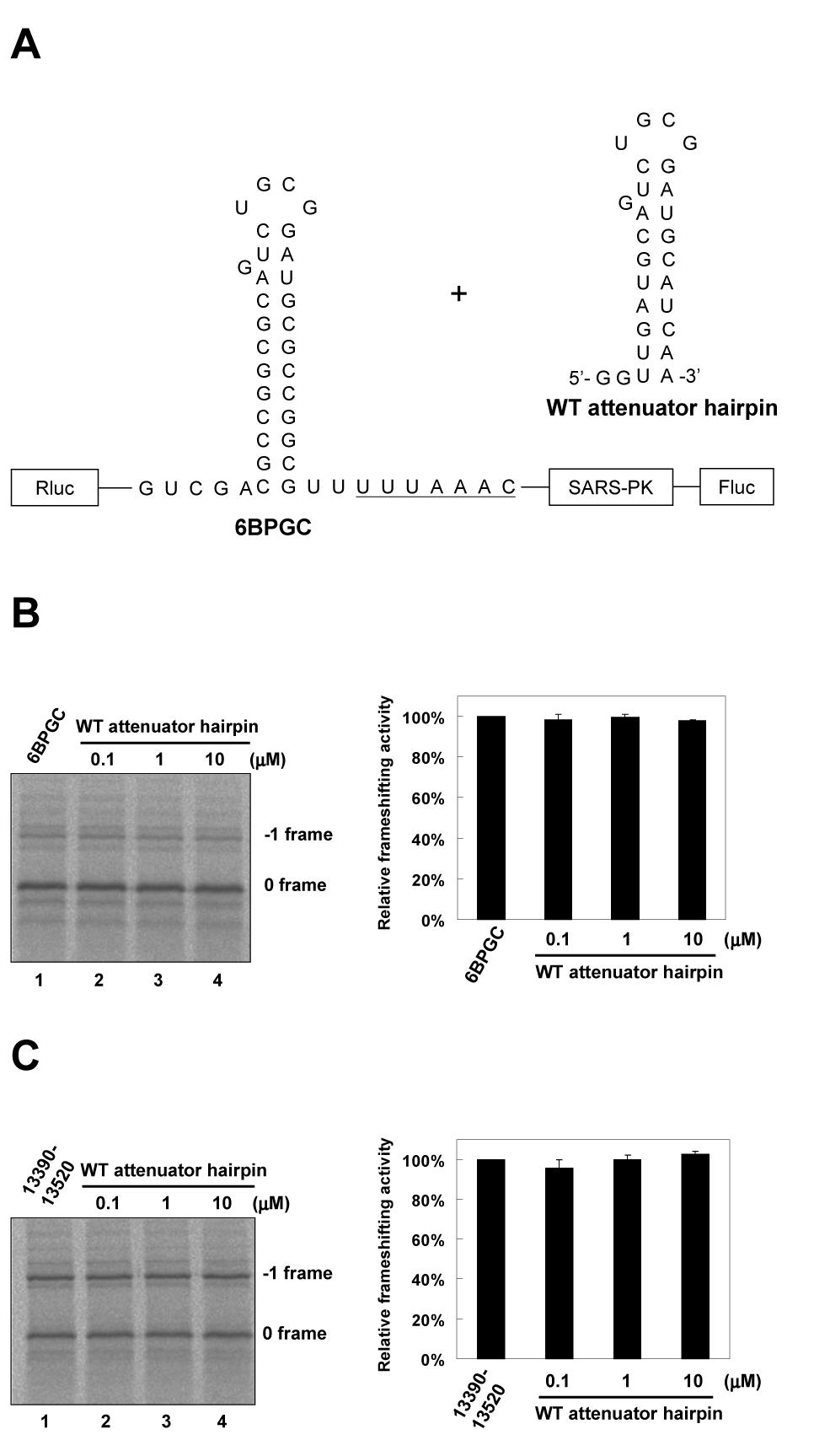

Supplement: Figure S3 — The -1 PRF efficiency of a reporter with or without an in-cis potent attenuator is not affected by titration of an attenuator RNA in-trans . (A) Schematic drawing of the reporter construct and the wild-type attenuator RNA hairpin used for in-trans titration. The SARS-PK was used as the stimulator in these -1 PRF reporters. (B) In vitro -1 PRF assays by SDS-PAGE analysis for the 6BPGC hairpin containing reporter in the presence of different amounts of in-trans WT attenuator hairpins (left), and the relative frameshifting activities in comparison with that of the reporter alone (right). The concentrations of the RNA hairpin are labeled as indicated. Error bars, s.d.; n = 3. (C) In vitro -1 PRF assays by SDS-PAGE analysis for attenuator-less reporter in the presence of different amounts of in-trans WT attenuator hairpins (left), and relative frameshifting activities in comparison with that of reporter alone (right). Error bars, s.d.; n = 3. (TIF) [file pone.0062283.s003.tif]

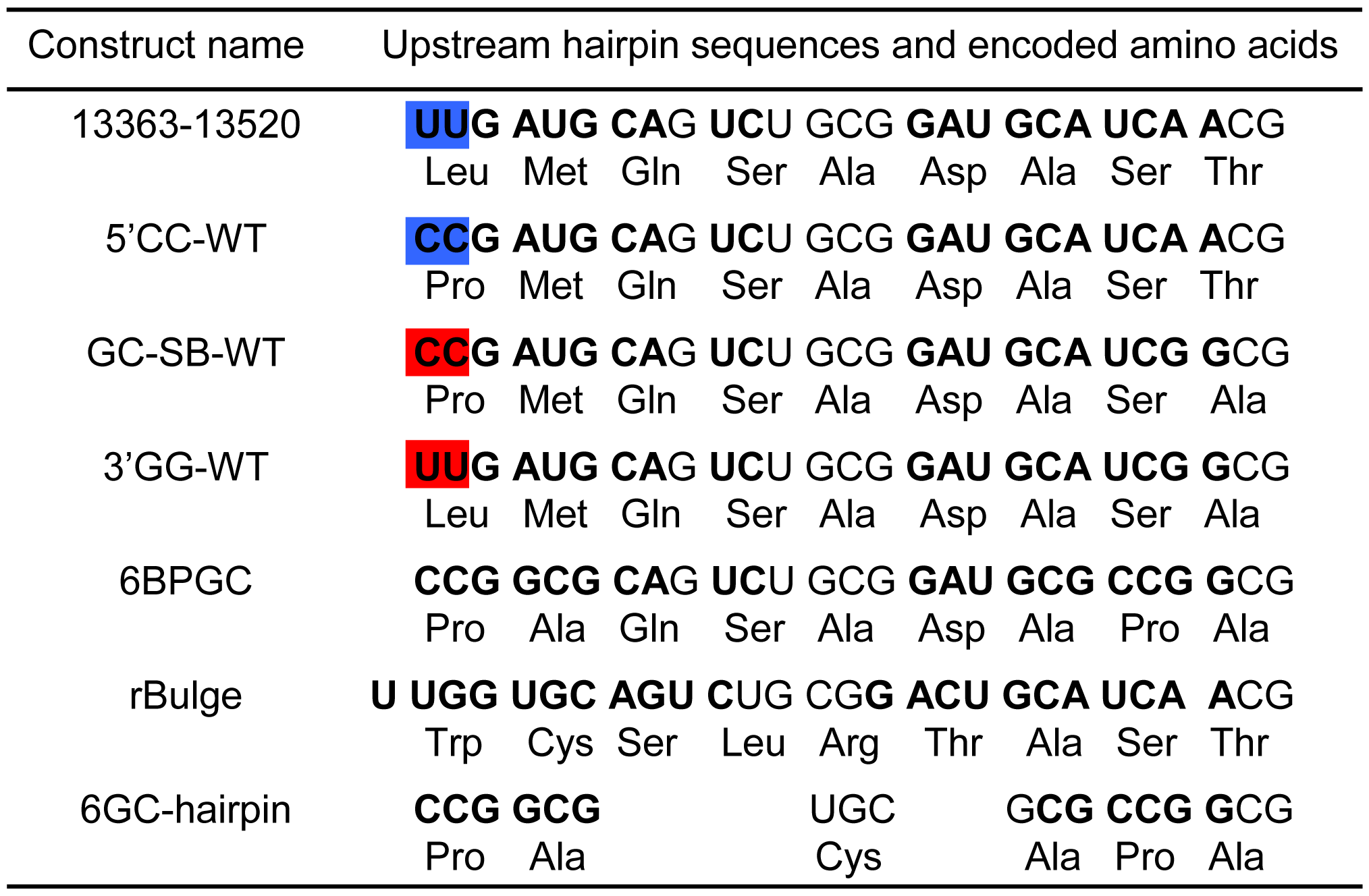

Supplement: Table S1 — The nucleotide sequences and encoded amino acids of selected upstream -1 PRF attenuator hairpin variants. The amino acids encoded by each 0-frame codon are shown below the codons, and the sequences corresponding to the 5′-half and 3′-half of each hairpin stem are boldly typed with the nucleotides involving particular base pairs disruption in two sets of constructs colored in red or blue. (TIFF) [file pone.0062283.s004.tif]
